# In vitro adaptation of *Plasmodium falciparum* reveal variations in cultivability

**DOI:** 10.1186/s12936-015-1053-0

**Published:** 2016-01-22

**Authors:** John White, Anjali Mascarenhas, Ligia Pereira, Rashmi Dash, Jayashri T. Walke, Pooja Gawas, Ambika Sharma, Suresh Kumar Manoharan, Jennifer L. Guler, Jennifer N. Maki, Ashwani Kumar, Jagadish Mahanta, Neena Valecha, Nagesh Dubhashi, Marina Vaz, Edwin Gomes, Laura Chery, Pradipsinh K. Rathod

**Affiliations:** Department of Chemistry, University of Washington, Seattle, WA 98195 USA; Department of Medicine, Goa Medical College and Hospital, Bambolim, 403202 Goa India; Department of Biology, University of Virginia, Charlottesville, VA 22904 USA; National Institute of Malaria Research (ICMR), Panaji, 403001 Goa India; Regional Medical Research Centre (NE), Dibrugarh, 786001 Assam India; National Institute of Malaria Research (ICMR), New Delhi, 110077 India

**Keywords:** Field isolate, Malaria patients, Cryopreservation, Culture

## Abstract

**Background:**

Culture-adapted *Plasmodium falciparum* parasites can offer deeper understanding of geographic variations in drug resistance, pathogenesis and immune evasion. To help ground population-based calculations and inferences from culture-adapted parasites, the complete range of parasites from a study area must be well represented in any collection. To this end, standardized adaptation methods and determinants of successful in vitro adaption were sought.

**Methods:**

Venous blood was collected from 33 *P. falciparum*-infected individuals at Goa Medical College and Hospital (Bambolim, Goa, India). Culture variables such as whole blood versus washed blood, heat-inactivated plasma versus Albumax, and different starting haematocrit levels were tested on fresh blood samples from patients. In vitro adaptation was considered successful when two four-fold or greater increases in parasitaemia were observed within, at most, 33 days of attempted culture. Subsequently, parasites from the same patients, which were originally cryopreserved following blood draw, were retested for adaptability for 45 days using identical host red blood cells (RBCs) and culture media.

**Results:**

At a new endemic area research site, ~65 % of tested patient samples, with varied patient history and clinical presentation, were successfully culture-adapted immediately after blood collection. Cultures set up at 1 % haematocrit and 0.5 % Albumax adapted most rapidly, but no single test condition was uniformly fatal to culture adaptation. Success was not limited by low patient parasitaemia nor by patient age. Some parasites emerged even after significant delays in sample processing and even after initiation of treatment with anti-malarials. When ‘day 0’ cryopreserved samples were retested in parallel many months later using identical host RBCs and media, speed to adaptation appeared to be an intrinsic property of the parasites collected from individual patients.

**Conclusions:**

Culture adaptation of *P. falciparum* in a field setting is formally shown to be robust. Parasites were found to have intrinsic variations in adaptability to culture conditions, with some lines requiring longer attempt periods for successful adaptation. Quantitative approaches described here can help describe phenotypic diversity of field parasite collections with precision. This is expected to improve population-based extrapolations of findings from field-derived fresh culture-adapted parasites to broader questions of public health importance.

## Background

Infections from *Plasmodium falciparum* remain a major public health threat around the world. While significant progress has been made in controlling malaria disease and deaths, recent estimates point to more than 3.2 billion people at risk for malaria, 200 million infections per year, and about 0.6 million deaths per year [1]. Among the most affected and most studied are children between the ages of two and 12 years in Africa, who account for the greatest number of deaths globally [2]. Beyond that, understanding variations in disease presentations and resistance to malaria counter-measures is of great interest around the world [3, 4], including in South Asia, which is understudied and offers interesting contrasts to malaria in Africa [5–9].

Culture-adapted malaria parasites can provide powerful information on the degree of threat presented from resistance to traditional drugs [10–16] and to artemisinin and its derivatives [17–24]. Culture-adapted parasites can also reveal modes of action of new anti-malarials [25–27] and physiological mechanisms by which parasites develop drug tolerance and ultimately become resistant [26, 28–30]. Gene expression in culture-adapted parasites has also been used to probe mechanisms of disease [30–34] and modes of action of protective antibodies [35–38]. Many basic studies of malaria parasites have been carried out on parasite lines collected decades ago [10–16]. With a resurgence of interest and increased investment in understanding the nature of malaria around the world [3–9], efforts to collect and characterize recent parasite isolates are also on the rise.

The value of phenotypic studies of culture-adapted malaria parasites depends, in part, on standardized methods to capture parasites and complete representation of parasite populations in culture-adapted collections. Since the original description of successful in vitro cultivation of *P. falciparum* parasites more than five decades ago [39, 40], many improvements and variations in methods and tools for culturing parasites have been reported, including the benefits of using Albumax over heat-inactivated (HI) plasma-supplemented media, the potential advantages of starting with lower haematocrit (HCT), and routine washing and processing of patient blood before adaptation [41–43].

Beyond the need for standardizing adaptation procedures, it is even more important to define what is considered adapted. In most studies, it is not always clear how much time investigators give a population of parasites to adapt and what fraction of naturally occurring variations in parasites are captured in the adapted culture. Even when parasites straight from the arm are initially visible in cell culture, it is not always clear which lines will truly proliferate successfully and indefinitely, and which lines will die off.

To understand the importance of experimental variables during culture adaptation, fresh incoming patient blood samples were divided into aliquots that were subjected to simple and manageable variations in culture adaptation. A number of variables were tested including initial high versus low haematocrit, initially washed versus unwashed infected blood from patients, and media supplementation with HI-plasma or with Albumax. By starting with many patient samples in a centralized hospital setting, it was possible to examine whether other variations affected culture adaptation. Such variations included initial parasitaemia, age of the donor patient, recent exposure to anti-malarials, and delays between sample collection and initiation of culture adaptation.

The results show that culture adaptation of freshly collected parasites is highly effective, particularly with 1 % haematocrit supplemented with 0.5 % Albumax. When working with HI-plasma, it was important to wash the incoming patient blood. Culture adaptation was occasionally forgiving of stresses such as delays between sample collection and processing and sometimes even recent drug treatment. Above all, successful cultivability and time to adaptation appear to be intrinsic properties of parasites. The present work frames successful experimental conditions for capturing representative new parasite lines in a field setting.

## Methods

### Ethical committees

This work was part of the Malaria Evolution in South Asia (MESA) programme project, a US NIH International Center of Excellence for Malaria Research (ICEMR). The study was approved by the ethics boards at Goa Medical College and Hospital, the University of Washington, and the Division of Microbiology and Infectious Diseases at the NIAID (US National Institutes of Health) as well as by the Government of India Health Ministry Screening Committee (HMSC).

### Patient isolates

Following informed consent, blood samples from *P. falciparum*-positive patients were collected in Acid Citrate Dextrose (ACD) (BD, India) vacutainers by MESA staff at Goa Medical College and Hospital (Bambolim, Goa, India) from April 2012 to January 2013. Enrolled patients ranged in age from 16 to 63 years old with a median and mean age of 26 and 32, respectively. MESA staff prepared Giemsa-stained thin and thick smears for parasitaemia determination and *Plasmodium* species identification. Rapid diagnostic test (RDTs) (Zephyr Biomedicals, Goa, India) were additionally used for the diagnosis of parasite species. One of 33 patients was co-infected with both *P. falciparum* and *Plasmodium vivax*. In two cases, patients were treated with an anti-malarial prior to a blood draw. The nature of the treatment and the time of treatment was available from the study site hospital records (see “Results”).

### Laboratory facilities at the hospital

The MESA-ICEMR has developed a Biosafety Level 2 cell culture facility one floor above the medicine wards of Goa Medical College and Hospital. To assure sterile manipulations, the on-site laboratory has 6 ft NuAire Nu-425-600S laminar flow cabinets and HEPA-filter air cleaners in the self-contained culture area as well as the corridors outside the culture area. The culture hoods are routinely tested for quality performance and used for culture-media preparations, patient blood sample processing, maintenance of parasite cultures, cryopreservation of cultures, and thawing of viable frozen parasites.

### Cell culture

Parasites were propagated with slight modifications of previously established culture methods [39, 40]. Human Type A+ fresh frozen plasma and human Type A+ red blood cells (RBCs) were purchased from the Rotary Blood Bank (New Delhi, India) from healthy individuals. RBCs were washed three times with RPMI medium under sterile conditions and subjected to quality tests (see below) prior to use with patient-derived parasite samples. Plasma was heat-inactivated by incubation at 56 °C for 30 min followed by centrifugation for 10 min at 1000×*g*. In a sterile environment, heat-inactivated plasma (HI-plasma) was directly added to RPMI-1640 at a 1:5 ratio to produce complete media. Albumax I and RPMI-1640 medium (HEPES, l-glutamine) were purchased from Life Technologies, and the final culture medium of 0.5 % Albumax I was further supplemented with 0.05 % hypoxanthine (Sigma-Aldrich).

Antibiotics were not added to the media. Antibiotics can potentially mask challenges in maintaining sterility and any drop in personnel performance in the culture facility. Antibiotics are not always specific and can also add potentially confounding variables to phenotypic and genotypic characterization of culture adapted parasites [44, 45]. It would be understandable if other investigators working under more challenging culture conditions find it helpful to use antibiotics in their media during culture adaptation.

### Quality control

HI-plasma and washed heterologous RBC used for culture media were regularly subjected to quality control to ensure their ability to support parasite growth. In brief, reference strain *P. falciparum* 3D7 was propagated with the newly prepared HI-plasma-supplemented media and washed RBCs for a minimum of two intra-erythrocytic cycles. Typically, five- to ten-fold growth was observed per 48-h invasion cycle. Patient isolates were randomly tested for mycoplasma contamination on a weekly basis.

### Variables for optimum culture conditions

As each sample arrived following venous blood draw to the on-site culture laboratory, it was aliquoted and subjected to eight different adaptation conditions.

To establish cultures under conditions 1–4 below, 200 µL of *unwashed* whole blood was seeded directly in 10 ml culture flasks under the following conditions (without additional external blood):

1. 1 % HCT, 20 % HI-plasma-supplemented RPMI-1640 media;
2. 1 % HCT, 0.5 % Albumax-supplemented RPMI-1640 media;
3. 0.25 % HCT, 20 % HI-plasma-supplemented RPMI-1640 media;
4. 0.25 % HCT, 0.5 % Albumax-supplemented RPMI-1640 media.

The remaining patient blood was first processed to remove plasma and white blood cell by centrifugation at 1000×*g* for 5 min and three washes with 10 mL of RPMI-1640 media. Cultures conditions 5–8 were initiated using 100 µL of packed, *washed* RBCs per flask with:

5. 1 % HCT, 20 % HI-plasma-supplemented RPMI-1640 media;
6. 1 % HCT, 0.5 % Albumax-supplemented RPMI-1640 media;
7. 0.25 % HCT, 20 % HI-plasma-supplemented RPMI-1640 media;
8. 0.25 % HCT, 0.5 % Albumax-supplemented RPMI-1640 media.

During culture maintenance, samples (5 µL) were removed from concentrated cells every 24–48 h to generate Giemsa-stained thin smears. Parasites were diluted with fresh media and uninfected RBCs as necessary to keep the parasitaemia below 1 % to prevent overgrowth.

### Adaptation from fresh blood draw

For a given patient isolate that showed parasites in culture, two of eight culture flasks were chosen for quantitative growth tests. The first flask that reached a 0.5–1.0 % parasitaemia threshold from two groups (HI-plasma-supplemented media and Albumax-supplemented media) was included in the present analysis. Culture 1 for Growth Test 1 was chosen from conditions 1, 3, 5, or 7 (HI-plasma-supplemented media, called Group 1). Culture 2 for Growth Test 1 was chosen from conditions 2, 4, 6, or 8 (Albumax-supplemented media, called Group 2).

### Tests for successful adaptation

Before formal growth tests began, when parasitaemia in a given patient-derived culture 1 or culture 2 reached >0.5 % for the first time, it was passaged to 0.25 % parasitaemia through the addition of the appropriate amount of fresh RBCs. For cultures with high initial patient parasitaemia (greater than 0.5 %), this first passage typically occurred on Day 1 or 2 to prevent initial overgrowth. Otherwise, the initial recovery to 0.5–1.0 % parasitaemia took as few as 8 days or as many as 30 days.

When parasitaemia first rebounded to 0.5–1.0 % parasitaemia, the culture was ready for Growth Test 1. This was initiated by reducing the parasitaemia to 0.25 % and determining parasitaemia 48 h later. Successful passage of Growth Test 1 required a four-fold increase in parasitaemia within a span of 48 h. Growth Test 2 was then immediately initiated by again reducing parasitaemia to 0.25 %. Successful passage of Growth Test 2 also required a minimum of four-fold increase in parasitaemia within 48 h. If a culture did not pass either Growth Test 1 or 2, a third growth test was conducted. Those cultures that failed multiple growth tests were tracked for a minimum of 35 days and then discontinued.

### Cryopreservation and thawing of samples

Unadapted parasites, were cryopreserved following venous blood draw and processing. Briefly, RBCs were spun at 1300×*g* and the supernatant media was removed. An equal volume of Glycerolyte 57 was added and solution was transferred to a cryovial. Long-term storage was under −80 °C or liquid nitrogen conditions. At later dates, the cryopreserved parasites were revived from −80 °C by palm thawing (~2 min), followed by transfer to a 15 mL centrifuge tube and drop-wise addition of one-fifth volume of 12 % NaCl. Ten mL of 1.6 % NaCl were added to the sample and this parasite solution was centrifuged at 1000×g for 5 min. The supernatant was removed and the RBC pellets were re-suspended with 10 mL of RPMI-1640. After a final centrifugation at 1000×*g* for 5 min, the thawed cells were introduced into culture with 10 mL of HI-plasma supplemented media at 2 % HCT. Maintenance and microscopic analysis was performed on each culture every 48 h. Growth tests were completed as discussed above.

### Genotyping of parasites

For an additional quality check of the present collections, which were largely made up of clonal parasites, DNA microsatellite analysis was used [46]. The analysis was first performed both on Day 0 samples, involving DNA preparations directly from the arm of the patient, and then again after adaptation to assure that adapted parasite genotypes in this low endemicity area were well represented in this collection, that there was no selective enriching of subpopulations of parasites from a few patients leaving other subpopulations behind, and most importantly that there was no cross-contamination between patient samples or from standard cell lines growing in the laboratory.

### Data capture and analysis

Demographic, diagnostic and clinical data were captured and managed using REDCap [47]. LabKey software was used for culture adaptation data storage, analysis and organization [48]. GraphPad Prism 6 was used for statistical analyses (unpaired t test and ANOVA) of the data presented in this report. The study data were available to partner laboratories in both India and the US in real-time.

## Results

### Parasite source

**Fig. 1:**
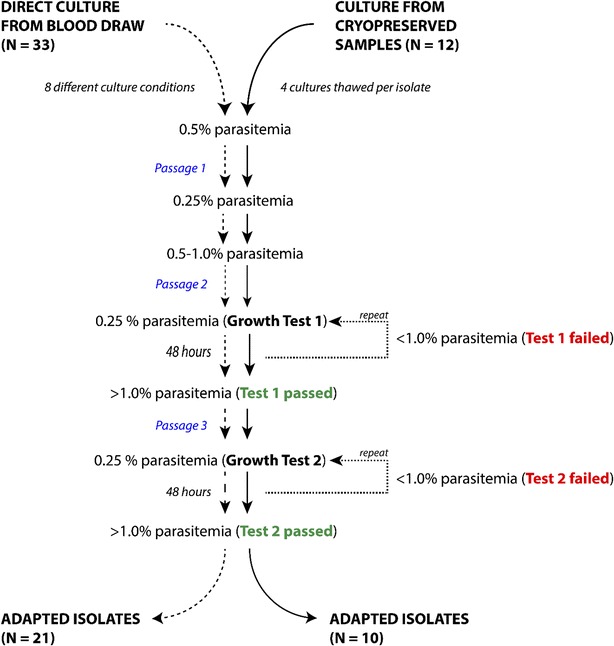
Workflow for testing adaptability of *Plasmodium falciparum* parasites from patients. Between 3 and 6 ml of blood was collected from each enrolled patient infected with *P. falciparum* or mixed co-infection with *P. vivax.* For direct culture (*left* path), aliquots of infected blood were used to establish 8 cultures under different conditions (See “Methods” and Table 1). For the adaptation from cryopreserved samples (*right* path), two independent researchers each thawed cryogenically preserved patient isolate samples in duplicate. When two cultures reached 0.5–1.0 %, two growth tests were conducted. Parasite cultures were maintained for as long as 35 days for adaptation from freshly drawn blood and as long as 45 days to allow adaptation from cryopreserved blood

Venous blood samples were collected from *Plasmodium*-positive individuals at Goa Medical College and Hospital, a tertiary care hospital with diverse malaria presentations, but no prior experience in laboratory-based parasite characterization. In addition to species determination in the hospital, all patients were retested for *P. falciparum* or mixed infection by repeat smear and RDT performed by MESA laboratory staff. The present study involved 33 patients infected with *P. falciparum*, one of which was a mixed infection with both *P. falciparum* and *P. vivax.* Each patient sample was used to seed and test eight flasks under different initial culture conditions (see “Methods” and Fig. 1). After that, all cultures were monitored for emerging new parasites every 24–48 h by light microscopy.

### Quantitative criteria for adaptation

In an analysis of fresh blood draw adaptations, out of 33 patient samples, 21 (~65 %) showed successful growth in at least one of the eight conditions tested (Table 1). Natural isolates of *P. falciparum* were considered adapted upon four-fold increases in parasitaemia over two 48-h time periods (see “Methods” and Fig. 1). This criteria was based on early experiences which suggested that a lower two-fold increase in parasitaemia was not a reliable predictable of stable future growth in culture and that a full ten-fold increase in growth took longer than was practical (unpublished data). To keep workloads manageable, the two cultures that grew fastest for each patient were subjected to the formal growth tests, but the two test flasks had to have two different media types: one with 20 % HI-plasma-supplemented media and another with 0.5 % Albumax-supplemented media.

**Table 1:** Most favorable culture conditions and time to adaptation of *Plasmodium falciparum* parasites, ‘straight from the arm’ of 33 patient samples (eight conditions tested per patient)

Culture ID	Initial parasitaemia (%)	Culture 1		Culture 2	
		Condition	Days to adapt	Condition	Days to adapt
CA1	0.2	2	9	6	Δ
CA2	1.1	2	10	5	10
CA3	1.9	2	11	1	18
CA4	2.6	2	11	3	11
CA5	2.7	2	13	5	13
CA6	13.5	2	16	NGT	–
CA7	0.7	2	18	8	18
CA8	3.8	8	20	7	Δ
CA9	0.0	2	22	1	Δ
CA10	0.5	3	21	5	Δ
CA11	3.6	4	12	1	23
CA12	6.0	5	13	8	Δ
CA13	2.6	7	22	5	Δ
CA14	1.9	6	9	6	Δ
CA15	0.1	6	15	1	Δ
CA16	3.3	5	16	6	19
CA17	9.7	6	18	8	Δ
CA18	0.9	6	20	1	22
CA19	1.3	6	20	2	Δ
CA20	9.2	6	23	8	Δ
CA21	0.4	7	33	NGT	–
NA1	0.4	NGT	–	NGT	–
NA2	0.2	NGT	–	NGT	–
NA3	0.4	NGT	–	NGT	–
NA4	0.6	NGT	–	NGT	–
NA5	0.0	NGT	–	NGT	–
NA6	0.0	NGT	–	NGT	–
NA7	3.6	NGT	–	NGT	–
NA8	2.0	NGT	–	NGT	–
NA9	0.5	2	Δ	NGT	–
NA10	1.6	NGT	–	NGT	–
NA11	2.0	6	Δ	NGT	–
NA12	1.7	5	Δ	7	Δ

Condition 1: Unwashed RBCs, HI-plasma, 1 % HCT

Condition 2: Unwashed RBCs, Albumax, 1 % HCT

Condition 3: Unwashed RBCs, HI-plasma, 0.25 % HCT

Condition 4: Unwashed RBCs, Albumax, 0.25 % HCT

Condition 5: Washed RBCS, HI-plasma, 1 % HCT

Condition 6: Washed RBCs, Albumax, 1 % HCT

Condition 7: Washed RBCs, HI-plasma, 0.25 % HCT

Condition 8: Washed RBCs, Albumax, 0.25 % HCT

Culture ID prefix CA- and NA- refer to ‘culture adapted’ and ‘non-culture adapted’, respectively

NGT refers to ‘no growth test’ was performed, as parasitaemia did not reach initiation milestones

Δ indicates that a culture did not pass growth tests during the 35 days of testing

### Most favorable media conditions

Table 1 shows the outcome of adaptation attempts from the 33 patient samples. Culture ID numbers CA1 to CA21 were assigned to patient isolates that were formally considered adapted. Culture ID numbers NA1 to NA12 were assigned to patient isolates that did not generate adapted parasites.

The aggregate data could be inspected with respect to: (1) how often a particular culture condition was represented in the most favorable adaptation protocol (conditions 1–8, see “Methods”); or, (2) how long it took for parasites to pass the formal growth tests. Of the parasite isolates that adapted, some conditions were clearly more favourable to rapid adaptation. Either condition 2 or condition 6 (both with 1 % HCT and 0.5 % Albumax) most frequently led to efficient culture adaptation. Eighteen out of 40 successful outcomes came from culture conditions having 1 % HCT and 0.5 % Albumax, when would expect ten out of 40 if all combinations of conditions were equally favourable for adaptation. With Albumax, it did not matter whether the infected whole blood was processed to remove white blood cells (WBCs) and HI-plasma (nine successes for condition 2 and eight for condition 6).

### From patient arm directly into culture

**Fig. 2:**
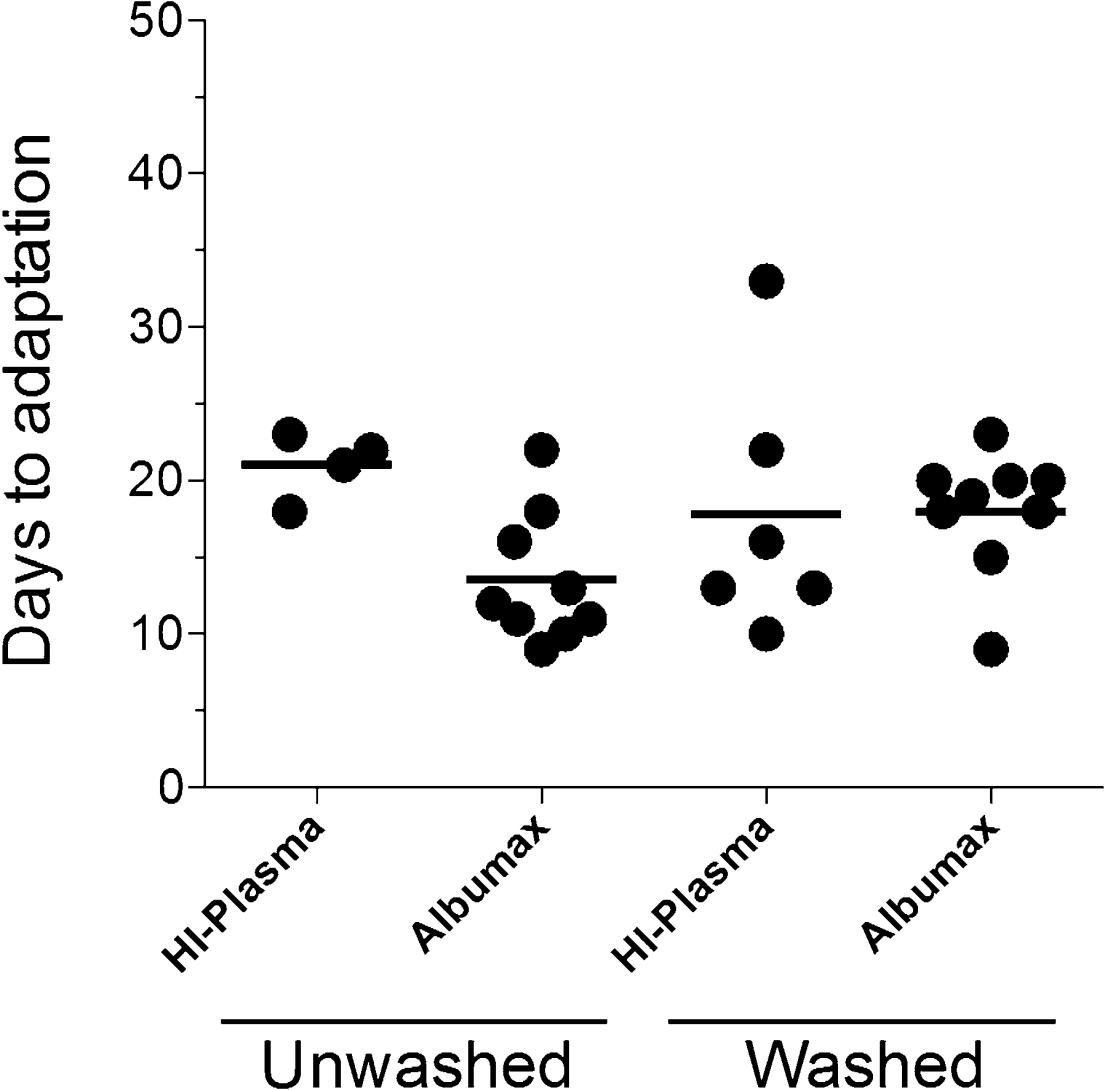
Time to adaptation is not affected by growth medium. For cultures initiated for adaptation from fresh blood draws, pairwise comparisons between all possible combinations of protocols showed no significant difference in time to adaptation between HI-plasma- vs Albumax-supplemented media nor between unwashed (whole blood) vs washed pRBCs (p > 0.1). All haematocrits are treated together in this data (Table 1)

With HI-plasma, condition 1 (unwashed infected blood transferred directly into culture at 1 % HCT with 20 % HI-plasma) and condition 3 (unwashed infected blood transferred directly into culture at 0.25 % HCT with 20 % HI-plasma) were highly under-represented in Table 1. This affirms that when it is important to use *unwashed* patient blood samples for culture adaptation in this setting, Albumax is desirable and HI-plasma presents a significant liability. This is most likely due to initial complement-mediated red cell lysis rather than effects of the medium on parasite growth since parasites adapted successfully in the presence of 20 % HI-plasma if the cells were washed before placing them in culture. It is likely that small diluted complement in plasma of unwashed parasitized patient blood was contributing to lysis when the medium had HI-plasma, but not when it had Albumax. A comparison of the time it took to pass the growth tests showed no difference between cells *washed* in HI-plasma versus cells *washed* in Albumax (Growth Test 1 and Growth Test 2, Fig. 2).

Each endemic setting often has to determine the type of media best suited for the particular research environment. For practical applications, in principle, Albumax-supplemented media may be advantageous because it is better defined than HI-plasma that comes from random in-country donors. The plasma from donors from an endemic region could potentially have varying levels of invasion blocking antibodies or even circulating anti-malarials. In addition, unlike Albumax, it can be difficult to obtain culture-quality, safe HI-plasma in some settings around the world. The ability of Albumax media to support minimally processed parasite containing RBCs, even direct whole blood, could further increase convenience especially when managing large numbers of samples under sterile conditions at a study site in an endemic setting. One could further argue that whole blood from a malaria patient as an inoculum for culturing parasites may be better because removal of extracellular components and human cells in human blood may hurt adaption by eliminating critical components for parasite growth. This is particularly true in light of recent new findings on exosome-like particles, which can facilitate cellular communications [49, 50]. Indeed, there are some indications that HI-plasma-supplemented media may be better suited for measuring invasion rates and phenotypes associated with virulence [51]. On the other hand, in the present setting, there may be advantages to washing infected RBCs and removing WBCs, inhibitory immune factors, or anti-malarials that interfere with growth. When adaptation occurred, time to adaptation was not significantly impacted by media type, RBC levels, nor washing of pRBCs (Fig. 2).

### Penalties from delayed processing

In the early days of the present project, due to some hospital collections late in the working week and some operational miscommunication on weekends, samples were unintendedly left out for long durations in ACD vacutainers at room temperature. Amongst these were patient samples NA1 and NA7, which experienced delayed entry into the culture system for 13 and 45 h, respectively, but no drug treatment. Delayed processing itself may potentially undermine cultivability. Based on this, hospital-based collections are now culture-adapted only when they arrive at the laboratory in a timely manner. Elsewhere in this NIH programme, at more remote locations, collections are kept on ice until they reach the appropriate laboratory for processing.

### Penalties from prior exposure to anti-malarials

Of the patient samples studied here, two enrolled patients out of 33 had been treated with anti-malarials for two to 15 h prior to blood collection by MESA staff and they offered surprisingly different outcomes in the cultivability tests. It was understandable why NA12 did not adapt to culture from fresh or from cryopreserved samples: the patient providing sample NA12 was treated with artesunate and primaquine for 14 h prior to blood draw. In contrast, patient sample CA21 had experienced artesunate and mefloquine exposure for 4 h prior to blood draw and the sample was not processed for an additional 16 h after collection. Surprisingly, the latter sample adapted using the two four-fold growth tests, though the culture was one of the slowest among the 21 isolates that adapted (about 30 days; Table 1). It is possible that during the early hours of drug treatment, a fraction of the population of drug-treated parasites goes into a dormant state that gradually comes back to a proliferative state over time [24]. This experience suggests that a complete collection of field samples could, and possibly should, include parasites from drug-treated patients because they could reveal mechanisms of dormancy. The fraction of parasite samples showing such tolerance for drug treatment and exposure to room temperature for an extended time in an ACD vacutainer may be small.

### Intrinsic variations in adaptability

**Fig. 3:**
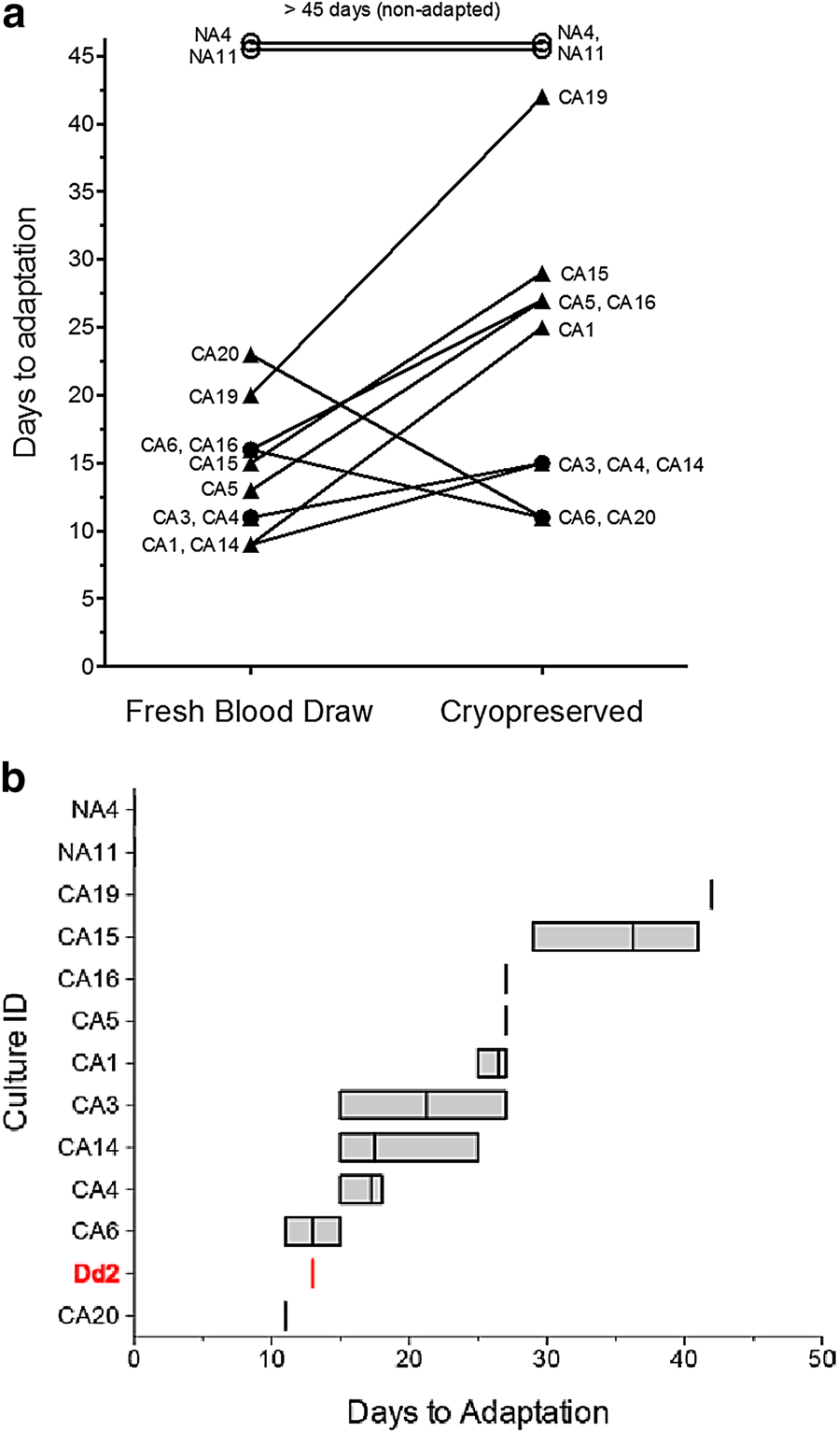
Time to adaptation varied predictively and reproducibly between different patient isolates. **a** A comparison of pairwise time-to-adaptation for parasites from direct blood draws versus from cryopreservation. Parasite samples that were subjected to immediate adaptation after a blood draw showed the same ranked-tendencies compared to cryopreserved immediately after blood draw from the patients, when tested together months later in identical media and identical host RBCs, the isolates showed roughly the same ranking in ease to adaptation. The fastest adapting parasites from some fresh patient blood draws (to the *left*) adapted as quickly as 8 days in culture (e.g., CA1 and CA14), others (e.g., CA15, CA16, CA19) took 15–20 days in culture, and yet others (NA4 and NA11) did not adapt at all. For this work, blinded, duplicate samples were adapted by each of two different scientists in the laboratory. Samples with a history of anti-malarial treatment or prolonged exposure to room temperature before sample processing were left out (see “Results”). **b** Cryopreserved parasites from individual patient isolates show large but reproducible variations in time to adaptation. For each cryopreserved patient parasite isolate, the vertical line inside a box represents the mean number of days to adaptation. The *box* around the vertical line represents the full spread of days to adaptation for that parasite isolate. The raw data is shown in Table 2

Freshly drawn blood placed in culture in a timely way and without drug exposure allowed parasites to adapt successfully, but at significantly different rates (Fig. 3a, left side). Overall, of the 33 isolates subjected to tests for ease of adaptability straight from the patient arm, two isolates did not adapt. Of the remaining 31, some isolates adapted as quickly as 8 days and some took as long as 23 days. This suggested that that there may be intrinsic variations amongst parasite isolates in adaptability to culture as previously projected [9, 30]. Of course, it was also possible that some parasite lines were better matched to the donor RBC or donor plasma in the media on the days they were adapted.

The possibility of intrinsic variations amongst parasite isolates to adapt to culture was tested differently: Overall, out of the original 33 patient-derived parasite samples tested earlier for adaptability straight from the patient arm, 12 cryopreserved samples were selected for testing for reproducibility of this potential trait. Of these 12 samples to be retested, 2 had not adapted straight from the arm, four had adapted very fast straight from the arm, and six had adapted at rates somewhere in between. In the retest, all 12 samples were allowed to adapt in parallel in the presence of identical host RBCs and identical culture media. The results showed that cryopreserved parasite isolates varied predictably in terms of the relative time required to adapt (Table 2; Fig. 3a, right side).

**Table 2:** Time to adaptation of ‘Day 0 cryopreserved blood’ with *Plasmodium falciparum* parasites thawed as duplicate replicates and maintained under HI-plasma, 2 % HCT conditions

Culture ID	Initial parasitaemia (%)	Time to adaptation (days)				
		Experimenter 1		Experimenter 2		Avg.
		Culture 1	Culture 2	Culture 3	Culture 4	
Dd2	1.9	13	x	13	x	13.0
CA1	0.2	25	27	27	27	26.5
CA3	1.9	18	15	25	27	21.3
CA4	2.6	15	18	18	18	17.3
CA5	2.7	27	Δ	Δ	Δ	27.0
CA6	13.5	11	13	13	15	13.0
CA14	1.9	15	15	15	25	17.5
CA15	0.1	29	41	34	41	36.3
CA16	3.3	Δ	27	27	27	27.0
CA19	1.3	42	Δ	Δ	Δ	42.0
CA20	9.2	11	11	11	11	11.0
NA4	0.6	Δ	Δ	Δ	Δ	**–**
NA11	1.0	Δ	Δ	Δ	Δ	**–**

Culture ID prefix CA- and NA- refer to ‘culture adapted’ and ‘non-culture adapted’, respectively

Δ indicates that a culture did not pass growth tests during the 45 days of testing. Dd2 is a highly drug resistant strain of *P. falciparum* derived from W2, that grows exceptionally well in culture

While adaptation from cryopreserved parasites was generally slower than earlier work from fresh parasites, the rank order of isolates for successful culture adaptability was very similar for 10 of the 12 retested parasite lines (Fig. 3a). Parasite lines NA4 and NA11, which did not adapt earlier, again did not adapt from the cryopreserved collection, even though the starting parasitaemia of 0.6 and 2 %, respectively, was higher than for many lines that adapted successfully (Tables 1, 2). Parasites CA19, CA15 and CA16, which adapted the slowest when placed directly into culture following collection, adapted the slowest after months of cryopreservation. Parasite lines CA3, CA4, CA14 and CA1, which adapted the fastest when placed directly into culture following collection, again adapted the fastest after months of cryopreservation. None of these samples, except CA21 (see above), were drug treated and all samples were processed within 1 h of collection. Thus, variations in culture adaptability again pointed to intrinsic properties of the parasites collected from patients.

### Reproducibility of variations in adaptability

The reproducibility of adaptation between the different patient-derived lines was excellent as was the rank order of the adaptability of the parasites preserved after months of cryopreservation. Table 2 and Fig. 3b show the variation in times to adaptation when a cryopreserved aliquot was placed in culture by two different scientists, each handling two original sample aliquots from the same patient isolate.

To illustrate the speed with which some cryopreserved patient lines adapted to culture for the first time, it is helpful to compare their recovery to that of the pre-adapted line Dd2 (in red). Figure 3b shows that lines CA20, CA6 and CA4 adapted to culture conditions for the first time in less than 15 days. Other lines, such as CA15 and CA19, took a month longer before they passed the formal growth tests.

There were two surprising outliers from the rank order patterns (Fig. 3a): patient isolates CA6 and CA20 adapted more rapidly after cryopreservation compared to parasitized RBCs straight from the arm. These two samples came from donor patients with the highest blood parasitaemia, 13.5 and 9.2 % respectively, in the sample set. It is possible that the washing steps in the process of preparing the cryopreserved parasites for culture helped remove some parasite-derived inhibitory factors in these blood samples with higher parasitaemia.

Overall, after accounting for samples with delayed processing and long drug exposure, patient-derived parasites were recovered from cryopreservation into culture >90 % of the time. In fact, comparing the ten patient samples that adapted straight from the arm to adaptation attempts from matched cryopreserved vials, the success rate was 100 %. This was very encouraging because it allowed the present field-based malaria parasite operations to decouple sample collection and cryopreservation from adaptation. The ability to store and move cryopreserved parasites facilitates phenotypic characterizations that are often conducted by different partners on the same cell lines at different times and even at different sites.

### Age of patients

**Fig. 4:**
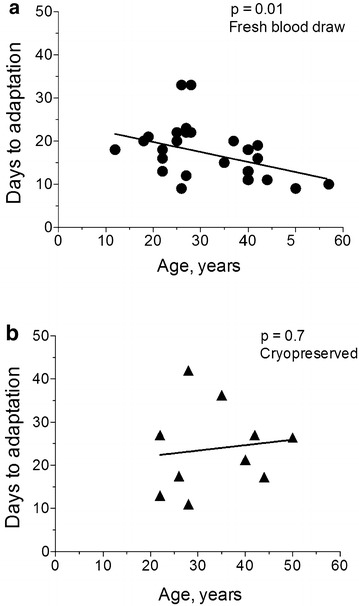
Time to adaptation was shorter in parasites derived from older patients than in parasites from younger donors. **a** Patient samples adapted straight from fresh blood draws showed faster time to adaptation in patients above the age of 32 than below the age of 32. **b** Patient samples adapted after cryopreservation had a less significant relationship between speed of adaptation and the age of the donor

In some malaria settings, age is associated with higher prior exposure to malaria parasite infections and increased protective immunity, as measured by the ability of antibodies to decrease RBC invasion [52]. It was hypothesized that stronger immunity in an older patient with *P. falciparum* could potentially select for parasites that use many different RBC invasion pathways efficiently and show fast growth in new cellular environments, such as first adaptation with donor blood. When culture adaptability of different parasites emerging from patients of different age groups was compared, a significant correlation was seen: parasites from older patients adapt to culture faster (Fig. 4, upper panel). This relationship is significant, but it did not hold when cryopreserved parasites, with greater scatter in the surviving parasites, were retested for adaptability (Fig. 4, lower panel). The overall longer adaptation times from cryopreserved parasites suggest that there was partial loss of viable parasites in cryopreserved samples, but it remains to be seen if there is disproportionate loss of parasite viability and cultivability in frozen samples from more aged patients. Overall, compared with age, intrinsic properties of parasites may contribute much more to the speed with which parasites adapt in culture.

### Parasitaemia of patients

It was possible that time to adaptation could be significantly affected by initial parasitaemia. Parasites from a patient, when placed in culture, have to deal quickly with acclimatizing to RBCs from a different donor and plasma from yet another donor. Adaptation may involve reconfiguring invasion processes to match initial culture conditions. Switch rates for invasion processes are related to numbers of parasites under selection [53]. It was hypothesized that a population of parasite cells at very low parasitaemia would not have significant variations in invasion ligands or pathways to overcome the challenges of adaption.

**Fig. 5:**
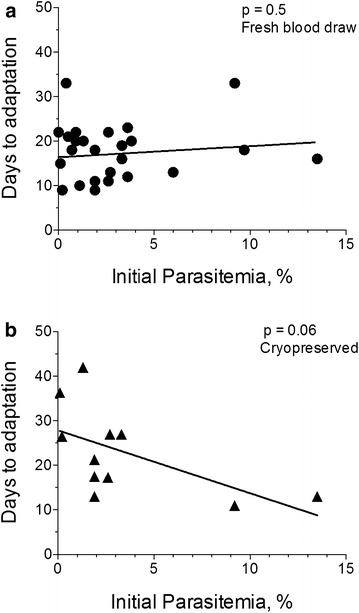
Time to adaptation was not related to patient parasitaemia. **a** Patient samples adapted from fresh blood draws showed roughly the same time to adaptation 8–22 days, even as parasitaemia ranged from <0.1 in 3 samples to >9 % in 3 samples. **b** After cryopreservation, low parasitaemia samples showed longer time to adaptation than fresh samples when starting parasitaemia was less than 2 %. This was presumably due to fragility of parasitized RBC. Recovery and adaptation was robust at parasitaemia above 2 %

No direct relationship was seen between initial parasitaemia and time to adaptation of a sample (Fig. 5, upper panel). In fact, sample CA9, in which parasitaemia was barely visible and incalculable by thin smear, adapted by day 22 or as quickly as some parasites that had as much as 100× higher starting parasites. Cryopreserved parasites with high parasitaemia showed a small, but significant, increase in adaptability (Fig. 5b). This appeared to be due to a decrease in viability of parasites at low parasitaemia during the cryopreservation process and, thus, poor adaptability from surviving cells. Overall, these data are consistent with the earlier indication that intrinsic properties of parasites may contribute the most to rapid adaptation in culture.

### Application to field operations

Based on these foundations, more streamlined procedures have been developed for adaptation of patient-derived parasites being collected at Goa Medical College and Hospital and at the other MESA-ICEMR field sites in South Asia. It is important to have a collection of parasites that has grown in HI-plasma conditions as well as Albumax conditions to minimize deviations from physiological conditions found in blood. The work flow utilizes two initial culture conditions: patient samples are routinely placed under culture condition 5 (washed, 1 % HCT, 20 % HI-plasma) and culture condition 6 (washed, 1 % HCT, 0.5 % Albumax) immediately after washing patient blood with RPMI 1640 medium under sterile conditions to remove WBCs and HI-plasma. While it would be easier to simply work under Culture Condition 2 (unwashed, 1 % HCT, 0.5 % Albumax), the laboratory facility at Goa Medical College and Hospital allows the luxury of washing every sample under sterile conditions for a variety of downstream applications and nested studies.

## Conclusions

In a first study of its kind, a formal definition of culture adaptation was established and these criteria were used to examine determinants of successful culture adaptation. Even in the first attempt at a new site, parasites from ~65 % of tested patient samples successfully adapted.

Amongst the successfully adapted parasite lines, there were large variations in time to adaptation. Although the cryopreserved lines adapted slightly more slowly, the rank order for time to successful adaptation was mostly preserved. The collective data suggest that intrinsic properties of parasites may play a major role in successful adaptation. Other variables, such as parasitaemia levels in a patient, did not influence the ability to adapt nor time to successful adaptation. Parasites derived from older patients, possibly with higher immunity, adapted significantly faster than parasites collected from younger patients.

The mechanisms underlying these variations in adaptability will be the focus of future detailed studies. In the meantime, the procedures adopted following this study are being used at multiple study sites of the MESA-ICEMR programme with larger sample sets and for genotypic and phenotypic characterization of field-derived parasite lines.

## Abbreviations

ACD: acid citrate dextrose
AS: artesunate
Cli: clindamycin
HCT: haematocrit
HI: heat-inactivated
ICEMR: International Centers of Excellence for Malaria Research
MESA: Malaria Evolution in South Asia
MQ: mefloquine
PQ: primaquine
pRBCs: parasitized red blood cells
